# Tracking SARS-CoV-2: Novel Trends and Diagnostic Strategies

**DOI:** 10.3390/diagnostics11111981

**Published:** 2021-10-26

**Authors:** Linda P. Guaman-Bautista, Erick Moreta-Urbano, Claudia G. Oña-Arias, Marbel Torres-Arias, Nikolaos C. Kyriakidis, Koray Malcı, Nestor Jonguitud-Borrego, Leonardo Rios-Solis, Espiridion Ramos-Martinez, Andrés López-Cortés, Carlos Barba-Ostria

**Affiliations:** 1 Centro de Investigación Biomédica, Facultad de Ciencias de la Salud Eugenio Espejo, Universidad UTE, Quito 170147, Ecuador; linda.guaman@gmail.com (L.P.G.-B.); erickmorett@gmail.com (E.M.-U.); claudia.sscc5@gmail.com (C.G.O.-A.); 2 Immunology and Virology Laboratory, Department of Life Science and Agriculture, Universidad de las Fuerzas Armadas, Quito 171103, Ecuador; mmtorres@espe.edu.ec; 3 Grupo de Investigación en Biotecnología Aplicada a Biomedicina (BIOMED), Universidad de Las Américas, Quito 170125, Ecuador; nikolaos.kyriakidis@udla.edu.ec; 4 One Health Research Group, Faculty of Medicine, Universidad de Las Américas (UDLA), Quito 170125, Ecuador; 5 Institute for Bioengineering, School of Engineering, University of Edinburgh, Edinburgh EH8 9LE, UK; koray.malci@ed.ac.uk (K.M.); N.Jonguitud@ed.ac.uk (N.J.-B.); leo.Rios@ed.ac.uk (L.R.-S.); 6 Centre for Synthetic and Systems Biology (SynthSys), University of Edinburgh, Edinburgh EH8 9LE, UK; 7 Experimental Medicine Research Unit, Facultad de Medicina, Universidad Nacional Autónoma de México, Mexico City 4510, Mexico; espiri77mx@yahoo.com; 8 Centro de Investigación Genética y Genómica, Facultad de Ciencias de la Salud Eugenio Espejo, Universidad UTE, Quito 170147, Ecuador; andresa.lopez@ute.edu.ec; 9 Escuela de Medicina, Colegio de Ciencias de la Salud Quito, Universidad San Francisco de Quito USFQ, Quito 170901, Ecuador

**Keywords:** COVID-19, nucleic acid amplification test, antigen testing, CRISPR-based diagnostics, nanotechnology-based diagnostics, automation

## Abstract

The COVID-19 pandemic has had an enormous impact on economies and health systems globally, therefore a top priority is the development of increasingly better diagnostic and surveillance alternatives to slow down the spread of the severe acute respiratory syndrome coronavirus 2 (SARS-CoV-2). In order to establish massive testing and contact tracing policies, it is crucial to have a clear view of the diagnostic options available and their principal advantages and drawbacks. Although classical molecular methods such as RT-qPCR are broadly used, diagnostic alternatives based on technologies such as LAMP, antigen, serological testing, or the application of novel technologies such as CRISPR-Cas for diagnostics, are also discussed. The present review also discusses the most important automation strategies employed to increase testing capability. Several serological-based diagnostic kits are presented, as well as novel nanotechnology-based diagnostic methods. In summary, this review provides a clear diagnostic landscape of the most relevant tools to track COVID-19.

## 1. Introduction

In late 2019, multiple cases of atypical pneumonia caused by a then unknown pathogen were reported in Wuhan, province of Hubei in China. The disease named COVID-19 quickly spread globally and, after considerable research efforts, the severe acute respiratory syndrome coronavirus 2 (SARS-CoV-2) was identified as the pathogen causing the disease [1]. Coronaviruses are a large group of viruses that can infect a wide range of vertebrate hosts [2]. Diseases caused by each of those viruses show different clinical manifestations, usually affecting the respiratory tract in humans and digestive disorders in other animals [3]. In the last 20 years, three highly pathogenic beta coronaviruses have emerged from zoonotic events and have caused human disease [4]. In February 2003, the severe acute respiratory syndrome-related coronavirus 1 (SARS-CoV-1) was first reported in Asia. According to the World Health Organization (WHO), a total of 8,098 people worldwide became infected and 774 died (global case–fatality ratio (CFR) of 11%) [5] across two dozen countries in North America, South America, Europe, and Asia [6]. Since September 2012, WHO has been notified of 2562 laboratory-confirmed cases of infection with Middle East respiratory syndrome-related coronavirus (MERS-CoV), with a global CFR of 34.4% since 2012 [7]. Finally, the current disease caused by SARS-CoV-2, to date, has resulted in 217,914,903 confirmed cases and 4,523,984 deaths in 223 affected countries, areas, and territories [8]. There have been many attempts to estimate the global CFR of COVID-19 [9,10,11,12,13,14], but it has proven challenging, mainly because different regions of the world are experiencing different stages of the pandemic [15] affecting those estimates.

SARS-CoV-2 belongs to the *Coronaviridae* family, subfamily *Orthocoronavirinae*, genus *Betacoronavirus*, subgenus *Sarbecovirus*, and the species severe acute respiratory syndrome-related coronavirus [16]. Coronaviruses are enveloped, positive-sense, single-stranded RNA viruses that are distributed broadly among humans, other mammalian, and avian hosts [17]. The genome of SARS CoV-2 (NCBI Reference Sequence: NC_045512.2) [18] is very similar to the genomes of other known SARS-CoV and SARS-related coronaviruses, including the one that caused the SARS epidemic in 2003 (SARS CoV, NCBI Reference sequence: NC_004718.3) [19] and other related coronaviruses (e.g., MERS-CoV) [20]. Therefore, studies of protein structure and function from those coronaviruses have been important for understanding the function and mechanisms of SARS-CoV-2 proteins [21]. In addition, recent structural and functional studies on SARS-CoV-2 proteins have provided critical knowledge of SARS-CoV-2 biology [17].

**Figure 1:**
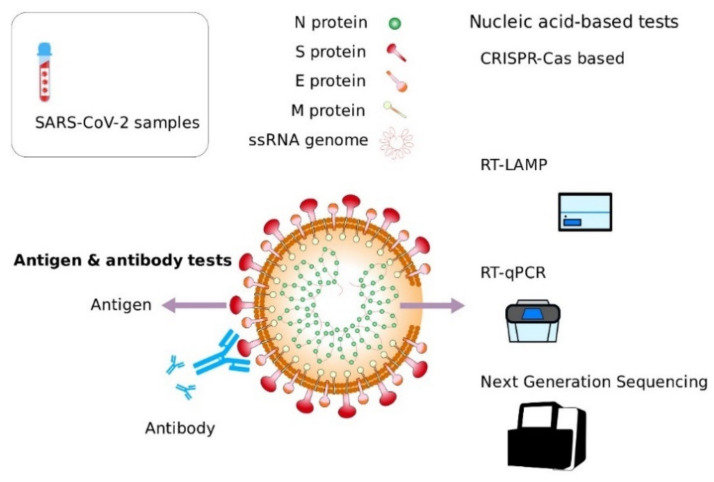
Structure of SARS-CoV-2 and diagnostic targets: SARS-CoV-2 structure has two components: viral genome (RNA) and viral proteins. Tests based on the detection of nucleic acids (RT-qPCR, RT-LAMP, CRISPR-Cas-based, NGS) target different regions of the viral RNA. Antigen tests aim at the detection of viral proteins such as spike protein (S). Serological tests detect host antibodies produced in response to SARS-COV-2 infection.

The SARS-CoV-2 genome is about 30 kb (Zhou et al., 2020), and it encodes at least 29 proteins, including 16 non-structural proteins (NSP), 4 structural proteins:(S), envelope (E), membrane (M), and nucleocapsid (N), and 9 accessory proteins (Figure 1). Some structural and non-structural proteins (S, E, M, N, 3CL protease, papain-like protease, RNA polymerase [22], and helicase) have been suggested as viable antiviral drug and diagnostic targets [23,24,25]. Although coronaviruses accessory proteins are frequently considered as non-critical for viral replication in vitro, some have an important role in virus-host interactions in vivo [26] and in vitro [27]. Different studies have shown that SARS-CoV-2 is a pathogen with a high efficiency in invading the cells of its host [28,29]. Currently, there is enough evidence supporting the preponderant role of angiotensin-converting enzyme 2 (ACE2) as a key factor allowing the binding and entry of SARS-CoV-2 to host cells [30,31,32].

## 2. Diagnosis

Due to the pandemic nature of COVID-19, testing has become an indispensable tool for monitoring the diagnosing and spread of the disease [33,34]. Multiple approaches and strategies for detecting SARS-CoV-2 have been developed. In this context, a clear understanding of the principles and differences between these strategies, their impact on the results, their correct interpretation, and the best conditions for their adequate use, is crucial [34]. In general, the major approaches for COVID-19 testing are: (1) detection of viral structural components (RNA and proteins) and (2) detection of molecules produced by the host immune system in response to SARS-CoV-2 infection.

RNA is a key structural component of SARS-CoV-2 and, given the previous development of several technologies allowing for the efficient isolation, amplification, and detection of specific nucleic acid sequences, the viral genome (RNA) is the most targeted molecule used for the detection of SARS-CoV-2. (Figure 1) [35,36]. This is the principle of widely used detection methods such as RT-qPCR, RT-LAMP, and next generation sequencing based assays, among others. A second group of methods have been developed targeting SARS-CoV-2 proteins in samples. These methods include viral antigen detection using proteomics-based tests and the nanosensor-based detection of viral proteins.

**Figure 2:**
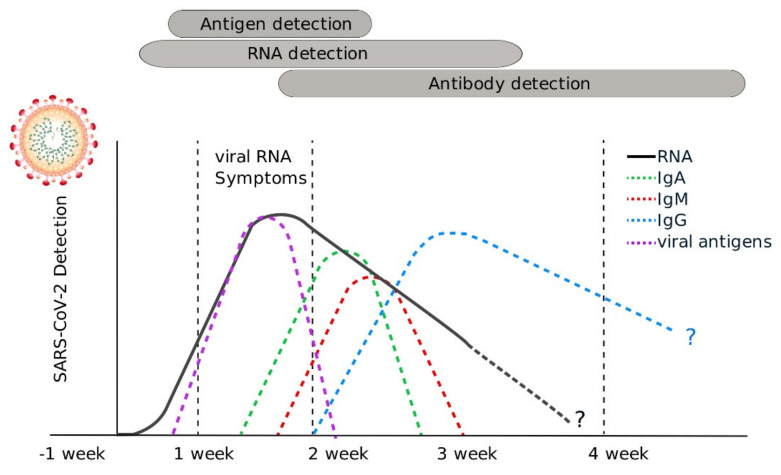
Timeline of SARS-CoV-2 detection using nucleic acid-based, antigen and serological tests: Detection of viral nucleic acids is useful from 5 to 15 days after the onset of symptoms. Because many nucleic acid detection tests include nucleic acid amplification, these tests can give positive results for prolonged periods. Due to the nature of antibody production and the variability of immune responses between individuals, serological tests can be used for seroprevalence studies and surveillance from the second week after the onset of symptoms. Antigen tests are useful for the detection of infected individuals from 5 to 15 days after the onset of symptoms.

On the other hand, the concentration of the different types of antibodies (IgM, IgG, IgA) produced by the host immune system in response to SARS-CoV-2 can be used as a tool for surveillance to better understand how much of the population has been infected with SARS-CoV-2, as well as how the virus is spreading through the population over time [37] (Figure 2). The detection of antibodies is the common principle of all serological tests.

It is important to highlight that although serological tests are very useful from an epidemiological perspective, given that antibodies can take several days or even weeks to be produced and may stay in your blood for several weeks or more after recovery, serological tests should not be used to diagnose COVID-19 and are not approved for the diagnosis of acute, active SARS-CoV-2 infection [38,39].

## 3. Nucleic Acid Amplification-Based Tests (NAAT) for Detection of SARS-CoV-2

Since the SARS-CoV-2 genomic sequence was made public in early January 2020, several primers, probes, and detection strategies have been developed and optimized worldwide (Table 1). Despite the increasing availability of assays, most strategies are based on the same basic principle: the accurate and sensitive identification of SARS-CoV-2 RNA sequences. As is further discussed in this section, the major differences of all those assays are in the method used to measure/detect the presence of viral genetic material.

### 3.1. Reverse Transcription-Polymerase Chain Reaction (RT-PCR)

RT-PCR is a widely used method in molecular biology and is considered the gold standard for the detection of SARS-CoV-2 [106] and other respiratory viruses [107,108,109]. Early detection of infection caused by SARS-CoV-2 by RT-PCR relies on the efficient detection of viral genomic sequences in clinical samples. Based on the established practices for other respiratory infections such as influenza, the nasopharyngeal swab has been widely adopted as the preferred sampling technique for SARS-CoV-2 along with other techniques such as saliva, oropharyngeal, nasal mid-turbinate, and nasal swabs [110]. However, some studies have also reported using blood, sputum, feces, and urine samples for SARS-CoV-2 detection [111,112]. 

Most of the described protocols for SARS-CoV-2 detection require the isolation of viral RNA from biological samples as a first step. RNA isolation must be performed in a careful and quick manner to avoid degradation caused by nucleases released from cells and present in the environment [113]. First, the sample is mixed with a lysis buffer to release the genetic material and preserve RNA integrity. The following steps are designed to eliminate DNA, protein, and other cell components in order to isolate the RNA by serial washes [113]. After RNA isolation, the next step for RT-PCR is the conversion of viral genome (RNA) into DNA. This process requires the specific binding of DNA primers to viral RNA sequences and the activity of an RNA-dependent DNA polymerase (reverse transcriptase) to generate a short complementary DNA copy (cDNA) of the viral RNA. In the real-time RT-PCR, the polymerase amplifies the viral cDNA, and the synthesis is monitored in real-time by using a fluorescent dye or a sequence-specific DNA probe labeled with a fluorescent molecule and a quencher molecule [114]. To date, most molecular diagnostic tests developed have targeted different SARS-CoV-2 genomic regions, including the ORF1b or ORF8 regions and the nucleocapsid (N), spike (S) protein, RNA-dependent RNA polymerase (RdRP), or envelope (E) genes, while the most-used genes employed as targets in diagnostic tests are N gene, ORF1b, ORF1a and the E gene [115].

Since the development of PCR in the 1980s, many assays used for detecting nucleic acid have been created, and a growing number of research and diagnostic laboratories around the world perform PCR routinely. The wide adoption of this method has been, and still is, critical because it allowed for the rapid implementation of RT-qPCR-based diagnostics facilities and increased the SARS-CoV-2 diagnostic capabilities in many regions. In addition, given that specific assays can be designed based on any DNA or RNA sequence, this technology is highly flexible and is relatively simple to apply to the detection of SARS-CoV-2 variants, and of course, for the detection of future infectious microbes. In a fashion similar to other nucleic acid detection methods, RT-qPCR-based detection heavily relies on the quantity, quality, and integrity of DNA or RNA in samples, therefore the development of simpler, cheaper, and more efficient sampling alternatives is a challenge for improving SARS-CoV-2 RT-qPCR assays.

### 3.2. Assays Based on Nucleic Acid Isothermal Amplification

Introduced in the early ‘90s, isothermal amplification offers an alternative to Polymerase Chain Reaction (PCR)-based detection methods [116,117]. In contrast to PCR, isothermal amplification can be performed without the need of thermocycling equipment. Instead, it can be carried at one reaction temperature and under relatively simple conditions (e.g., in a water bath) [118]. More than ten types of isothermal amplification methods have been developed [119]. Some are based on DNA replication, enzyme-free nucleic acid assembly, and the use of enzymes for digestion [118]. In this review, we focus on six different techniques, and, where specified, examples are given for its application in COVID-19 diagnostics.

Isothermal amplification detection tests have become popular because they can be performed with simpler laboratory equipment and produce results in shorter times than conventional RT-qPCR assays. Despite this, significant challenges should be addressed to fully exploit this testing technology as a massive and frequent SARS-CoV-2 (and other infectious agents) detection tool, including: (i) reducing the number of micropipetting steps needed and (ii) avoiding the need of cold chain conditions to transport or store reagents.

### 3.3. Nucleic Acid Sequence-Based Amplification (NASBA)

Self-sustained sequence replication, better known as Nucleic Acid Sequence-Based Amplification (NASBA) was originally designed for RNA amplification [120]. In this technique, RNA hybridizes to the forward primer for the synthesis of its complementary chain to obtain cDNA using RNAase H. The newly synthesized cDNA contains a promoter region for the formation of an intermediate double-stranded (ds) cDNA, which is formed by using a second primer [121]. Several copies of antisense RNA strands to the target RNA are produced when T7 DNA-dependent RNA polymerase carries out transcription from the dsDNA. Then, the antisense RNA and cDNA become templates for the continuous cycle carried out by reverse transcriptase. The reaction can happen at 41 °C and it usually takes 1.5 to 2 h to complete. Colorimetric assays, gel electrophoresis, and real-time fluorescence can be used for amplicon detection [118]. This method has been used in combination with next generation sequencing (NGS) for the detection of SARS-CoV-2 [121].

### 3.4. Loop-Mediated Isothermal Amplification (LAMP)

Loop-Mediated Isothermal Amplification or LAMP is a low-cost diagnostic technique which relies on the activity of a strand-displacement DNA polymerase and four to six primers to exponentially amplify the target sequence [122]. This method amplifies nucleic acid under isothermal conditions at about 65 °C [123]. The forward inner primer (FIP) and the backward inner primer (BIP) are crucial to the amplification. They both contain two functional sequences: the first one for priming at an early stage and the second for self-priming at a later stage. The functional sequences described for FIP and BIP correspond to the target sequence of the target DNA. The result of the amplification is stem-loop DNA that can be detected by real-time assays [118]. 

As a diagnostic method, LAMP has several advantages, such as avoiding the need of a thermal cycler, making LAMP simpler and cheaper [124]. Moreover, due to the use of several primers, this technique has a high specificity, allowing it to discriminate a single nucleotide difference. Besides, the amplification is highly efficient (DNA can be amplified 10^9^–10^10^ times in 15–60 min) [125]. In addition, LAMP is fast due to the activity of the strand-displacement polymerase eliminating the need for a denaturation step, allowing the amplification and detection to occur in a single step. Additionally, in the case of low-resource settings, the amplification can be monitored by using a cheap turbid meter and, after, the addition of an intercalating agent [126]. LAMP has been used previously as a tool to diagnose diseases such as malaria [122] and tuberculosis [127].

In the context of COVID-19, RT-LAMP is the technology used by several assays of different companies (Table 1) worldwide. In addition, tests based on RT-LAMP have been approved by the FDA, CDC, and other regulatory agencies in many countries [128]. RT-LAMP has proven to be effective in detecting viral RNA, showing high specificity and sensitivity, thus being a potentially robust technique during the global COVID-19 pandemic [129].

In addition to its technical advantages for diagnostics, LAMP is a versatile method that has been coupled to other strategies to improve diagnostics. A clear example of this is STOP (SHERLOCK Testing in One Pot), a CRISPR -based method for the diagnosis of SARS-CoV-2 [130]. This method relies on the amplification performed by LAMP with further detection using CRISPR-Cas12 [130]. This is a very simple test that can be carried out at room temperature and, as the name implies, in a single pot.

### 3.5. Rolling Circle Amplification (RCA)

RCA is a simple and efficient amplification method that relies on the activity of highly processive DNA or RNA strand-displacing polymerases to generate a long single-stranded DNA (ssDNA) or RNA molecule [131]. RCA has attracted attention for diagnostics because of its simplicity and capability of being performed at a constant low temperature (room temperature to 37 °C) in a relatively short time (approximately 90 min). Importantly, due to its molecular principle, RCA avoids the generation of false-positive results, a common problem of other detection assays. This method is based the use of a circular probe (C-probe) formed by the interaction and subsequent ligation of the analyzed sequence with the 5′ and 3′ ends of a single-stranded DNA probe [132]. This C-probe hybridizes with the primer, which is elongated by the strand-displacement polymerase, resulting in the exponential amplification of the target DNA or RNA. It must be mentioned that DNA ligase catalyzes the ligation of the C-probe with the target only in the case of perfect complementarity of the 3′-and 5′-end sequences [133] avoiding false-positives [134].

RCA has been used before for detecting single nucleotide polymorphisms [135,136], *M. tuberculosis* genomic DNA [137], SARS-CoV [138], Ebola, and other pathogens [139]. Recently, a study described a modified RCA assay capable of detecting up to picomolar concentrations of synthetic viral DNA strands of SARS-CoV-2, Influenza A (H1N1pdm09), and Influenza B [140]. As in other nucleic acid amplification methods, RCA output can be detected by measuring fluorescence, by using colorimetric chemistry, or by coupling the results with other readout techniques [139].

### 3.6. Transcription-Mediated Amplification (TMA)

TMA is a technology based on isothermal amplification, that can be performed in a single tube [141] to detect target RNA sequences. This technology is more efficient than RT-PCR [141], as it can produce 10 billion amplicons in 15–30 min [142] and it does not require a thermal cycler [142,143], making TMA a more affordable alternative.

TMA employs two primers: one of them contains the promoter sequence for RNA-polymerase. The first step of the process consists of the hybridization of the primer–promoter to the target RNA. Then, a reverse transcriptase creates a copy of DNA based on the RNA target by extension. Later, an RNAse H degrades the RNA in the resulting RNA–DNA duplex. The second primer binds to the DNA copy, and the reverse transcriptase creates a new strand of DNA, resulting in a double-stranded DNA molecule. Then, an RNA polymerase initiates the transcription, since it recognizes the promoter sequence in DNA [142]. The detection is conducted by the HPA separation/detection procedure. First, the amplicon is hybridized with acridinium ester-labeled DNA probes. Then, a chemical reaction separates the hybridized probes from the unhybridized probes. The final step consists of the addition of the reagents into the reaction tubes to produce the chemiluminescent signal, which is measured by a luminometer [142].

TMA is an interesting alternative that is being used to diagnose COVID-19 [144] with a sensitivity of 98.15%, compared to RT-qPCR (96.25%) with a detection limit of 5.5 × 10^2^ copies in 1 of 5 samples [145]. These results included 116 nasopharyngeal swabs [144,145]. The Aptima SARS-CoV-2 assay consists of target capture, TMA, and Dual Kinetic Assay (DKA). In the first step, the target RNA is isolated by capturing oligomers which have a complementary sequence to the target and a string of deoxyadenosine residues. Then, the oligomers:target complex is then captured out of the solution by decreasing the temperature of the reaction to room temperature. The deoxyadenosine region covalently attaches to magnetic microparticles which contain poly-deoxythymidine molecules. The microparticles are pulled to the side of the reaction tube by magnets. Then, the supernatant is aspirated, and the particles are washed. After the target is captured, the sample passes to TMA. This assay amplifies and detects two conserved regions of the ORF1ab gene in a single reaction [146]. The detection step is mediated by DKA detection technology, which is based on HPA. However, DKA uses two different acridinium ester molecules in two different nucleic acid probes [142].

### 3.7. Recombinase Polymerase Amplification (RPA)

Another polymerase-based amplification method is the recombinase polymerase amplification (RPA). This technology is an isothermal amplification method developed by Piepenburg et al., in 2006 by using proteins involved in recombination, synthesis, and DNA repair [147]. The mechanism in which this method works starts with a recombinase that binds to oligonucleotides in the presence of ATP and high molecular polyethylene glycol (also referred as crowding agent). This mixture forms a recombinase–primer complex which screens the template DNA until a homologous sequence is found. Then, a primer stop occurs so that the primer interacts with the template while single-stranded binding proteins stabilize the displaced DNA strand in order to prevent it from ejecting the complex from the target DNA. Finally, the recombinase breaks away and a polymerase binds to the 3′ end of the primer and starts to synthesize a new strand of DNA in the presence of dNTPs [148]. If this process is repeated in several cycles, linear (with one primer) and exponential (with a forward and a reverse primer) amplification can be achieved [147,149].

Certain aspects must be taken into account in the design/usage of RPA components. Primers used in RPA were thought to be 30–35 bases in length, however some studies have reported that PCR primers (≈19–21 mers) can be used, and that successful amplification can be achieved [143,150,151]. Additionally, 5-end guanines should be avoided while 3-end cytidines and guanines improve amplification performance, while GC content should be between 30% and 70%. Although RPA can work at a range of temperatures from 22–45 °C, optimal temperatures tend to be in the range of 37–42 °C [152,153], and multiple equipment can be employed to regulate the temperature such as incubators, heating blocks, or even body heat, facilitating its use as a point-of-care test in the field [154]. RPA can successfully amplify dsDNA, ssDNA, cDNA, and methylated DNA from a large variety of organisms such as viruses, bacteria, fungi, animals, and plants. Moreover, RPA has proven to be effective in different sample types such as body fluids and animal products [148].

Two main advantages of RPA over PCR have been pointed out by various studies: reaction temperature and reaction time. The latter is notorious if we compare the 15–40 min run time of RPA with the usual run times that PCR takes to show results [149]. Additionally, several detection methods have been developed to visualize results in RPA, such as solid phase RPA combined with electrochemical, ring resonators, and fluorescent and colorimetric detection [148,155,156].

Although RPA has been studied and used for the detection of various viral and bacterial pathogens, it appears to be confined to research-only activities for now [132]. Ebola detection by RPA studies has suggested that the diagnosis with recombinant polymerase amplification can be deployed in coming Ebola outbreaks due to the low amount of equipment required by this technique: a heating block and a centrifuge [157]. Some studies have used RPA as a step in CRISPR diagnostic systems, while RPA as the main detection technique has also been tested for COVID-19 [158,159].

## 4. Next Generation Sequencing (NGS) for Detection of SARS-CoV-2

First introduced as Massively Parallel Signature Sequencing (MPSS), next generation sequencing has been in the market since around 2004 [160]. The COVIDSeq Test is the first NGS test approved by the US Food and Drug Administration for COVID-19 diagnostics [161]. This test is designed for RNA detection of SARS-CoV-2 in nasopharyngeal, oropharyngeal, and mid-turbinate nasal swabs with a capacity of up to 3072 samples to be processed in 12 h [102]. 

Although NGS has proven to be a useful detection method, as in other diagnostic methods, as in the case of other diagnostic strategies, the test should not be used as a diagnostic strategy by itself, as results from the NGS test need to be confirmed with features such as clinical observation, patient history, and epidemiological information [102]. A critical disadvantage of NGS limiting its broad application for COVID diagnostics is the high cost of reagents and materials needed and the requirement of complex laboratory equipment [162,163].

## 5. CRISPR-Based Tests

Since the early days of the pandemic, the high transmission rate of SARS-CoV-2, as well as the high frequency of asymptomatic, but still infectious, cases, took the world by surprise. Even more than a year after the first cases, the lack of a high-throughput, high sensitivity, simple and low-cost diagnostic test still limits the massive detection of the positive individuals, inhibiting not only early medical intervention but also allowing for further disease transmission [164].

Rapid and reliable diagnostic challenges can be tackled by new-generation Clustered Regularly Interspaced Short Palindromic Repeats (CRISPR)-based platforms that offer fast pathogen-tailored detection assays, as well as the possibility to simultaneously detect hundreds of pathogens per individual in a robust multiplex fashion [165] as a point-of-care molecular diagnostic test [166].

Clustered Regularly Interspaced Short Palindromic Repeats are a group of DNA sequences found in bacteria and archaea which serve as an immune system or an immune “hard drive” [167]. Although this immune hard drive was first discovered more than three decades ago [168], it was not until 2012 that their potential as genetic editing scalpels for prokaryotic, eukaryotic, and even mammalian cells was appreciated [169,170]. Therefore, it comes as no surprise that, after 2012, a group of academic laboratories working in collaboration with pharma counterparts leverage CRISPR-Cas systems to develop the DNA Endonuclease-Targeted CRISPR Trans Reporter (DETECTR) [171] and Specific High Sensitivity Enzymatic Reporter UnLOCKing (SHERLOCK) [172] developed the first pathogen diagnostic platforms based on this novel technology.

The DETECTR system uses “off-the-shelf” reagents, portable heat blocks, and a lateral flow readout, similar to pregnancy tests, which can achieve a point-of-care setup that can detect HPV16 and HPV18 in patient samples within 1 h [171]. The reported sensitivity of DETECTR assay is 95%, with a specificity close to 100% [171,173]. Due to the simple principle of targeting Cas proteins, CRISPR-Cas detection assays can be easily programmed to detect practically any pathogen by modifying the guide DNA or RNA that recognizes and directs the Cas enzyme to a specific sequence of the pathogen’s genetic material, including coronaviruses [171,174].

In a recent study, a research group from UCSF reported the design and validation of a SARS-CoV-2 DETECTR diagnostic assay [175]. As in other nucleic acid-based diagnostics, the assay’s initial steps are comprised of nasopharyngeal or oropharyngeal swab sampling and the ensuring of RNA extraction and a one-step reverse transcription and loop-amplification of viral genetic material (RT–LAMP) with primers that target the E and N genes of SARS-CoV-2 [141]. Then, the CRISPR step involves incubation, with Cas12-guide RNAs targeting sequences found in three SARS-like coronaviruses (SARS-CoV-2, bat-SL-CoVZC45, and SARS-CoV), and the detection of target sequences inducing a conformational change and cleavage by lbCas12a enzyme [175]. Activated lbCas12a starts cleaving nearby single-stranded DNA nonspecifically, including the fluorescent-quencher probe, releasing the fluorophore and increasing the fluorescence in the final readout [176]. The limit of detection of this method is reported to be around 10 copies per μL of reaction [175]. According to the authors, the assay showed no cross-reaction with other coronaviruses or influenza strains and showed a lower sensitivity than RT-qPCR; nine out of eleven RT-qPCR positive confirmed samples were positive using this assay [177,178]. Furthermore, the authors carried out an additional step, running 30 RT-qPCR positive and 30 negative samples, and demonstrated a positive predictive value of 95% and a negative predictive value of 100% for the SARS-CoV-2 DETECTR assay [175].

An alternative CRISPR-based detection method named All-In-One-Dual CRISPR-Cas12a (AIOD-CRISPR) was recently developed. The method uses a dual guide RNA approach to augment specificality and in an isothermic, one-pot amplification; the CRISPR readout reaction enables its use in a point-of-care setup, delivering results in 40, 20, or 1 min depending on the target and conditions [179]. As a proof of concept, HIV and SARS-CoV-2 RNA derived from plasmids were detected at a load of 1.2 and 4.6 copies/µL, respectively [180]. The method was further evaluated by detecting HIV RNA extracted from human plasma samples, exhibiting a performance comparable to RT-qPCR tests [180]. A study has used AIOD-CRISPR to test clinical swabs and the results showed to be consistent with the RT-qPCR methods approved by the CDC [179,181].

In 2017, a group of scientists from MIT and Broad Institute reported the development of Specific High Sensitivity Enzymatic Reporter UnLOCKing (SHERLOCK) [182]. This CRISPR-based technique for the detection of pathogens uses a similar approach as the DETECTR, while harnessing Cas13a and Cas13b (instead of Cas12a) enzymatic activity to produce a detectable fluorescence as a readout [182]. Cas13 orthologs recognize single-strand RNA sequences and exhibit a promiscuous RNase activity detected by including a fluorochrome-quencher probe. This method has been previously used to detect specific strains of Zika and Dengue virus to distinguish pathogenic bacteria, genotype human DNA, and to identify cell-free tumor DNA mutations [172].

Recently, Zhang and colleagues have presented a detailed SHERLOCK-based protocol for detecting SARS-CoV-2, starting with RNA purified from patient samples [158]. According to the presented data, the test can detect SARS-CoV-2 target sequences at a concentration of 10–100 copies per microliter of input within 60 min. In addition, once RNA has been extracted, the test can be performed using a dipstick without requiring elaborate instrumentation [158]. Currently, there is a SARS-CoV-2 SHERLOCK detection kit, as indicated in Table 1. Additionally, a high-throughput platform called CARMEN-Cas13 was developed by Pardis Sabeti’s lab. CARMEN-Cas13 simultaneously differentiates all 169 human-associated viruses, with at least 10 published genome sequences including SARS-CoV-2 [183]. Using a similar approach, the same lab developed a CRISPR-based surveillance tool for the detection of 67 viral species and subspecies, including SARS-CoV-2, phylogenetically related viruses, and viruses with similar clinical presentation [184]. This protocol has been tested with clinical samples [185].

Another CRISPR-based SARS-CoV-2 detection method worth mentioning is Cas13-based, Rugged, Equitable, Scalable Testing (CREST). CREST was designed to address three of the main hurdles—reagent accessibility, equipment availability, and cost—that limit the scalability of Cas13-based testing particularly in low-resource areas [186]. The rationale of CREST is using affordable reagents and equipment for RNA extraction, amplification, and detection. Final output is displayed in low-cost and easy-to-use fluorescent visualizers. According to the authors, the sensitivity of CREST is equivalent to RT-qPCR. This method has been successfully used as a rapid SARS-CoV-2 surveillance tool in asymptomatic college students [186].

Cas-based detection depends heavily on the quality of the sample and the integrity and quality of the extracted RNA. In addition, due to the involvement of various enzymatic steps, the robustness of the method still has room for improvement. To massively deploy sensitive, reliable, and easy-to-use Cas-based detection assays, it is important to optimize clinical performance and enhance the robustness of the assays. Given the simple principle of nucleic acid detection by Cas-based methods detection, it is expected that the next generations of SARS-CoV-2 tests will show enhanced clinical performance and will be optimized for the detection, not only of the original SARS-CoV-2 sequences, but of novel variants in the population as well.

## 6. Serology-Based Tests

Since the early days of epidemiology, serological tests have been a very useful tool to assess previous infections and track the community spreading of diseases [187], including SARS-CoV-2 [188]. In this context, serological tests can be useful for (1) estimating epidemiological variables such as the degree of transmission in the community and its burden [189], (2) performing mass testing to reduce the exposure of susceptible individuals to the virus [190], and (3) identifying individuals with a strong immune response to the virus and whose isolated antibodies can, potentially, be used for a convalescent plasma trial [4,191].

Several antibody detection platforms have been developed over the last 30 years, including enzyme-linked immunosorbent (ELISA) assays and lateral-flow assays [192]. Serological tests are designed to detect antibodies produced by the host in response to specific pathogens, in this case SARS-CoV-2, and, ideally, to minimize cross-reactivity to antibodies generated in response to other viruses or bacterial pathogens [193]. However, given the nature of the antigen–antibody interactions and the structural similarities between antigens from related viruses, cross-reactivity may exist.

Although the most common serologic tests available measure antibodies that bind to viral S and N proteins, not all of these antibodies are considered neutralizing/protective, therefore results from these kind of tests should not be used for identifying individuals who are protected against SARS-CoV-2 infection. In general, serological tests have lower sensitivity and specificity than average RT-qPCR-based tests. Negative results should be interpreted with caution, as negative serology does not definitively rule out acute infection [194].

Serologic assays for SARS-CoV-2 are currently being cleared by the U.S. Food and Drug Administration (FDA) for emergency use and are available and include indirect or lateral flow ELISA assays [195].

During the current pandemic, serological tests have been important for detecting the previous infection of SARS-CoV-2 and became popular because some are cheaper and simple to use. Despite this, they should not be used to substitute molecular testing for detecting acute infections. Some of the advantages of this kind of test are its low-price and simplicity to use. To improve SARS-CoV-2 serological testing, the main challenges are improving the sensitivity and specificity of the assays to enhance its clinical performance. Table 2 shows some serological tests developed to detect SARS-CoV-2.

### Antigen Testing

Antigen testing is a type of immunoassay designed to detect the presence of one or more specific proteins of the pathogen, allowing the crucial identification of infected individuals (symptomatic or asymptomatic), so that they can receive the appropriate care, follow public health measures, such as self-isolation, and so that contact tracing can be implemented without delay [226].

In the case of COVID-19, The FDA has issued an EUA for the first antigen assay, the Sofia 2 SARS Antigen FIA test, which employs immunofluorescence-based lateral flow technology in a sandwich design used to detect viral nucleocapsid protein in nasopharyngeal and nasal swab samples with test results within 15 min [227].

Antigen tests may not be as accurate as molecular tests, but they are more accessible in terms of availability and ease of use, and can be used to amplify tests without laboratories, including frequent retesting if necessary. Modeling studies have shown that an availability of results within 15–20 min and the frequency of testing are more important than sensitivity [226].

SARS-CoV-2 antigen tests developed during 2020 have comparable clinical performance to RT-qPCR [228], being useful for the detection of symptomatic and asymptomatic individuals [227]. Moreover, since antigen testing is significantly cheaper (5 to 10 times cheaper than the average RT-qPCR test) [229], simpler, and faster to use (approximately 15 min) than other diagnostic tests, this technology has become a powerful tool for reducing the spread of COVID-19, particularly in community settings such as universities, schools, and offices [230]. Moreover, a frequent method of antigen testing has been implemented as a safe way to reopen economies while controlling the spread [226]. In a recent study, Chaimayo et al., demonstrated that SARS-CoV-2 antigen detection (Standard™ Q COVID-19 Ag kit) shows a sensitivity (98.33%; 95% CI, 91.06–99.96%) and specificity (98.73%; 95% CI, 97.06–99.59%) comparable with the real-time RT-PCR assay [228]. Another advantage of antigen testing is that this type of test is the most likely to detect a positive person when at the peak of their infection, where people are most able to transmit the virus to others.

Regarding its use, WHO [8] has suggested the direct detection of SARS-CoV-2 viral proteins by nasal swabs and other respiratory secretions by lateral flow immunoassay, giving results in less than 30 min, as they offer the possibility of rapid, inexpensive, and early detection of the most infectious cases of COVID-19 in suitable settings. At the time of revision of this article, 13 tests have been granted Emergency Use Authorization from the FDA [231] (Table 3).

## 7. Nanotechnology for COVID-19 Diagnostics

The synthesis of nanoparticles (NPs), the development of nanosensors and microchips, and the use of devices have become essential tools for the diagnosis of various diseases, including COVID-19 [244]. In recent months, an increasing number of studies have shown that nucleic acid sequences can be detected and play an essential role in diagnosis. However, the detection of single nucleotide variations, such as polymorphisms or small nucleotide changes in viral variants, has proven to be challenging [245].

**Figure 3:**
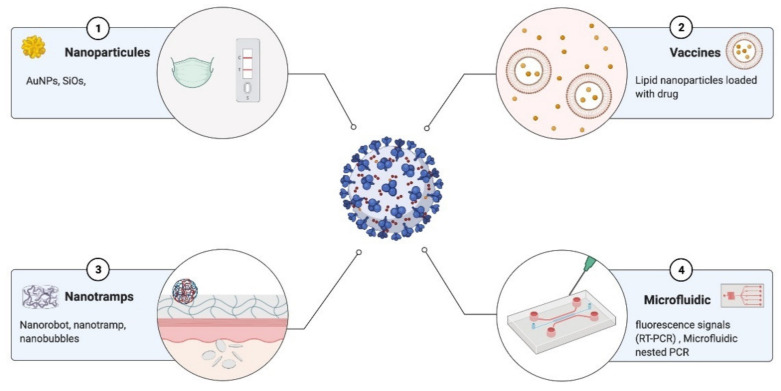
Nanomaterial tools for fighting future pandemics: (**1**) diagnostics, (**2**) vaccine delivery, (**3**) cell tramps, and (**4**) microfluidics are the future to fight against the pandemic. Created with BioRender.com (accessed date 15 January 2021).

A particularly relevant technology useful for pathogen and antibody detection purposes are gold NP-based immunoassays. In these assays, antibodies are coupled to gold NPs that, in the presence of a viral antigen or antibody, form a tertiary complex, leading to the immobilization and aggregation of the complex [246]. Other examples of the use of nanotechnology are: Biosensors based on graphene field effect transistors (FETs) coupled to a specific antibody against SARS-CoV-2 spike protein [247]; Dual-function plasmonic biosensors combining plasmonic photothermal effect (PPT) and localized surface plasmon resonance to detect Au nanomaterials coupled to complementary DNA sequences to detect SARS-CoV-2 hybridized cDNA [248]; Dendrimer-like DNA nanostructures (DL-DNA) for DNA detection (Li Y) system-on-a-chip (SoC) technology, whose detection mechanism is based on RNA aptamer conjugated to QDs [249] and materials such as silica that have been used to identify bioimaging and biodetection, and pathogen detection [250] (Figure 3).

In addition, gold nanoparticles coated with sulfated ligands, silver nanoparticles, hybrid silver, and copper nanoparticles can bind to the HIV envelope glycoprotein gp120 and inhibit HIV-1 infection in vitro in cell models [251]. Single wall carbon nanotubes (SWCNT) were also proposed as antiviral carriers, testing the increase in antiviral activity of hybrid materials compared to that of antiviral drugs [252]. Even antiviral and antibacterial textiles were based on nanomaterials; however, the commercialization of new functional nanomaterials is still not clear yet, and seems to be limited by nanotoxicology concerns or various other practical aspects (off-target and/or any other unpredictable effects, costs, performance production, durability, environmental impact, etc.). The emergence of new diseases has generated increased development and innovative tools in various areas of biotechnology and nanomedicine. The development of Au nanoparticles has mainly been useful for detecting different coronaviruses and respiratory infections, as presented in Table 4.

## 8. High-Throughput and Automated Screening for COVID-19

Given that test, track, and trace are crucial to tackle the COVID-19 pandemic, these processes are the core recommendation by CDC, European Centre for Disease Prevention and Control (ECDC), WHO, and other health agencies [262]. The rapid spread of COVID-19 has led to a huge demand for diagnostic screening [263], and to meet this goal while increasing the testing capacity of cities and countries, automated workflows have become key to enable high-throughput screening at an increased speed while improving diagnostic precision and reducing labor costs [264].

Several alliances between academic laboratories and companies such as Analytik Jena, Beckman Coulter, Hamilton, Tecan, Roche, Opentrons, among others, have focused their time and efforts in an impressive way to provide automated testing solutions for COVID-19 diagnosis [265]. These automated solutions were designed to cater to the various alternatives of molecular diagnosis protocols, such as the SHERLOCK [130] and LAMP [266] assays, although the RT-qPCR assay is still considered the “gold standard” for automated for SARS-CoV-2 testing [267].

### 8.1. Automation Platforms for SARS-CoV-2 Testing

**Figure 4:**
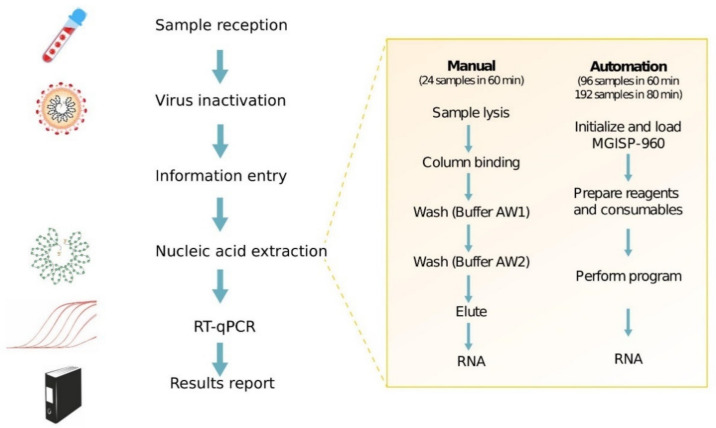
General RT-qPCR test workflow and comparison of manual and automated nucleic acid extraction in Huo-Yan Lab. The use of MGISP-960 platform to automate nucleic acid extraction increased the testing capacity, simplified the steps for extraction, and reduced the time per test. Adapted from [146,269].

An impressive example of automation support for COVID-19 testing and screening was the Huo-Yan Air Laboratory or “Fire Eye Lab’’ built in Wuhan, where the COVID-19 outbreak started in China. The Huo-Yan Lab uses automated nucleic acid extraction as a part of the RT-qPCR testing workflow, reaching 14.000 testing capacity per day on February 9 in a 2000 m^2^ site. This testing capacity was further increased to 20.000 per day by March 1 [268]. To achieve this, MGISP-960 platforms produced by the Chinese biotech company, MGI, were used to automate RNA extraction using the MGIEasy Magnetic Beads Virus DNA/RNA Extraction Kit to achieve high-throughput and standardized clinical testing, as shown in Figure 4 [81,269].

Manual RNA extractions using the QIAamp Viral RNA Mini Kit were compared to the platform automation, showing that the manual RNA extraction of 24 samples took on average 1 h and 50 min, whereas the automated extraction could finish processing 192 samples in 1 h and 8 min with a single run. The Ct values between the manual automated extraction resulted in no significant statistical difference, which was necessary to validate the automated platform [269].

In parallel to Huo-Yan Laboratory and MGI automated alternatives, several other biotech companies focusing on automation platforms rapidly adapted their technologies for COVID-19 screening. For instance, Hamilton offered an automated RNA extraction solution with its MagEx STARlet platform, enabling it to process 96 samples simultaneously [270]. Moreover, this platform is compatible with five commercially available magnetic bead-based RNA extraction kits, including MagMAX™ Viral/Pathogen NA Extraction Kit (Thermo Fisher, Waltham, MA, USA), Quick-DNA/RNA™ Viral MagBead Kit (Zymo Research, Irvine, CA, USA), and the Maxwell^®^ HT Viral TNA Kit (Promega, Madison, WI, USA). In addition to RNA extraction, PCR Prep STARlet for automated qPCR mix prep solution is also offered by the company as a compatible automation platform with FDA-approved kits such as TaqPath COVID-19 Combo Kit (Thermo Fisher), 2019-nCoV Kit, (Integrated DNA Technologies, Coralville, IA, USA), and the 2019-nCoV CDC Probe and Primer Kit (BioSearch Technologies, Hoddesdon, UK) [270].

The popular Fluent and Freedom EVO platforms of Tecan were adapted for use in automated RNA extraction and PCR preparation processes [271]. A Freedom EVO platform allowing for a fully automated qPCR reaction set-up works in compatibility with the real-time thermocyclers of Thermo Fisher, Bio-Rad, and Roche [271].

These technologies are currently being improved at a very rapid scale through innovative partnerships such as the collaboration of Zymo Research, focusing on molecular biology tools with Tecan and Opentrons to optimize the magnetic bead-based automated RNA extraction process [272,273,274,275].

### 8.2. Biofoundries Role against COVID-19

Biofoundries, which are non-profit organizations with state-of-art automation platforms and high-throughput equipment focusing on accelerating and prototyping biological designs for bioengineering applications [276]. Such facilities were uniquely positioned to contribute to the development of novel automated platforms for SARS-CoV-2.

**Figure 5:**
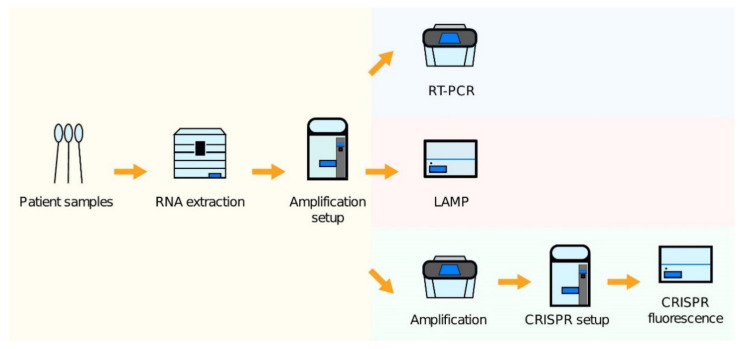
Schematic illustrations of three different diagnostic workflows from patient samples: A thermal cycler device is necessary in the case of RT-qPCR tests, while plate reader or other alternatives are available for measuring the readout in the case of CRISPR-Cas and LAMP tests. Adapted from [277].

The London Biofoundry configured its existing automated liquid handling infrastructure to COVID-19 screening platforms using three different workflows, encompassing RT-qPCR, CRISPR-Cas, and LAMP detection methods [276]. These three methods were automated and validated using various liquid handling platforms [276]. Figure 5 illustrates the automated workflows for the three different SARS-CoV-2 assays [276]. 

To test the platform performance, the researchers also tested two different RNA extraction kits from Analytik Jena and Promega and three different qPCR master mixes from ThermoFisher (TaqPath and Fast Virus) and NEB (Luna). Ct values of 10^3^ copies/mL of virus-like particles cwere observed around 30 cycles, while Ct values of 10^4^ copies/mL of VLP were approximately 27 cycles in both RNA extraction kits. For the LAMP workflow, however, it was observed that at least 42.5 copies of VLP were necessary for appropriate detection. The RT-qPCR workflow was validated with 173 patient samples obtained from the North West London Pathology using two different RNA extraction kits, obtaining an adequate R^2^ correlation of 0.8310 and R^2^ = 0.9357 for the innuPREP Virus DNA/RNA Kit (Analytik Jena) and Maxwell HT Viral RNA extraction kit (Promega), respectively. This automated platform employing the RT-qPCR workflow became operational in two London hospitals from May 2020 with a 2000 testing capacity per day [276].

There are also commercially available alternatives from several suppliers such as Hologic and Roche. For example, the Cobas^®^ 8800 (Roche, Basel, Switzerland) platform can process 1056 samples in eight hours or 384 samples in 4 h [59]. On the other hand, Panther Fusion from Hologic can run 336 samples simultaneously when 28 cartridges are used at the same time [278]. However, these platforms are also relatively expensive, and they mainly use proprietary reagents and cartridges which are tailored to their own platforms.

### 8.3. Free and Open Source Scientific and Medical Hardware (FOSH)

Even though the combination of the FeliX and Echo 525 were proven to be effective automation platforms for SARS-CoV-2, their accessibility by many laboratories is limited due to the higher costs of the equipment. To counteract this, the free open-source hardware and software community has played a prominent role for COVID-19 diagnostics, contributing to ease the burden on the global health systems. This has been achieved by the development and sharing of tools freely available for the community for further study, customization, and commercialization at a lower cost [279]. The best example of this for automation is Opentrons’ platform OT-2, which is an affordable and open-source lab automation system allowing to scale-up and automate hundreds of assays and workflows.

The OT-2 has gained popularity in the scientific community day by day, thanks to its flexibility and affordability [280]. While the Felix and Echo 525 cost are in the order of hundreds of thousands of pounds, the customizable OT-2 costs between 5 and 15 thousand, depending on the selected configuration.

**Figure 6:**
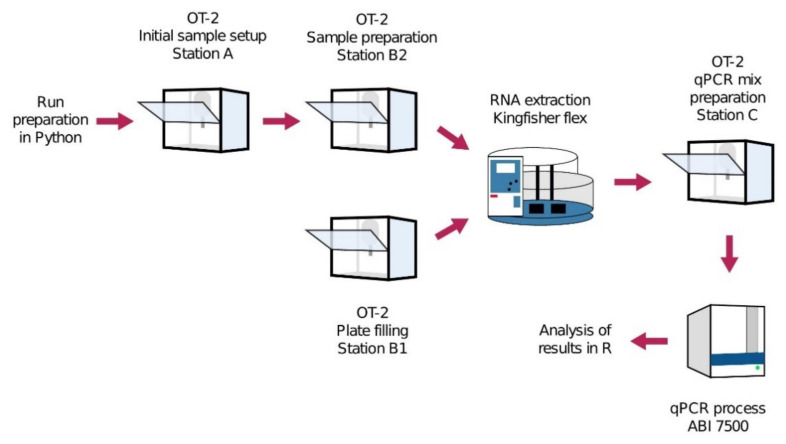
ROBOCOV circuit: Run preparation is done by using open-source Python codes. Initial sample setup, sample preparation, and plate filling are performed by OT-2 Station A, B1, and B2, respectively. RNA extraction is processed by KingFisher Flex. Then, qPCR mic preparation is done by OT-2 Station C, and, finally, the qPCR process is run by ABI 7500. The analysis results are exported as a user-friendly R file. Adapted from [280].

Researchers at the Biomedical Diagnostic Center (CBD) of the Hospital Clinic of Barcelona have developed an automated COVID-19 screening circuit named ROBOCOV, using four OT-2 robots from Opentrons, a KingFisher Flex extraction instrument from ThermoFisher, and an ABI 7500 qPCR device [280]. In this circuit, the initial setup, sample preparation, plate filling for RNA extraction, and qPCR mix preparation steps were carried out by four OT-2 robots while RNA extraction was performed by the KingFisher Flex and real-time qPCR process was done by the ABI 7500 qPCR device, as shown in Figure 6.

This study run preparation was done by using open-source Python codes. Initial sample setup, sample preparation, and plate filling are performed by OT-2 Station A, B1, and B2, respectively. RNA extraction is processed by KingFisher Flex [280]. Then, qPCR mic preparation is done by OT-2 Station C, and, finally, the qPCR process is run by ABI 7500 [280]. The analysis results are exported as a user-friendly R file. Due to this parallel and automated molecular testing circuit consisting of six platforms, the researchers tram obtained a high screening capacity completing the testing process of 96 samples in 4 h while starting each new run every 70 min. This set-up was further assessed and validated by the European Molecular Genetics Quality Network, which provides external quality assessment, and the findings revealed a comparable performance and consistent Ct levels compared with other validated automated platforms from Roche and Hamilton. A similar ROBOCOV is currently being used for high-throughput COVID-19 screening as an FDA-approved method at the Biomedical Diagnostic Center of the Hospital Clinic of Barcelona, with a 2400 test capacity per day [281]. The CLIAHUB facility, which intends to aid on COVID-19 testing in the San Francisco Bay Area, have also demonstrated that research institutions can contribute to COVID-19 testing and that the workflow provided by their report can process a vast number of tests by using eight automated liquid handles into the RNA extraction step [282].

The studies mentioned in this section highlight the key role that the automation of molecular diagnosis has had in the scaling and speeding up of COVID-19 testing and screening. It also showcases the need to develop novel and affordable automated diagnostic workflows to be deployed in lower income settings where testing is currently done predominantly manually. The automated platforms developed during the COVID-19 outbreak are very relevant, as they can easily be adapted for diagnostics for many other types of biological threats and infectious diseases, allowing the global community to be better prepared for future pandemics.

## 9. Conclusions

The COVID-19 pandemic has been and continues to be a threat to economies and health systems worldwide. Currently, no standard treatment for COVID-19 is globally available. Despite the enormous efforts that have allowed the unprecedented development of multiple highly effective vaccines against COVID-19 in record time, the broad access and distribution of vaccines in many countries is still a significant challenge. Therefore, the implementation of efficient and versatile COVID-19 testing policies remains a key strategy to tackle the effects of COVID-19, to its epidemiological features, and to prevent further damage from this virus and from additional SARS-CoV-2 variants.

Given the structure of the SARS-CoV-2 virus, and due to the previous development of technologies for the detection of nucleic acids and proteins, the major diagnostic strategies for COVID-19 are based on the detection of these molecules. The main objective of this review is to present a clear and useful diagnostic landscape from the most common strategies and technologies to novel diagnostic alternatives, describing their principles, as well as highlighting their advantages and disadvantages. It is also intended to review some of the most relevant automation platforms useful for boosting diagnostics capabilities, as well as diagnostic alternatives that are based on the application of nanotechnology principles.

## Figures and Tables

**Table 1:** Diagnostic tests based on detection of nucleic acids.

Test Name	Manufacture Country	Technology	LoD	Sample	Approval	Ref
1copy COVID-19 qPCR Multi Kit	Korea 1 drop	RT-qPCR	0.2 copies/µL	Nasopharyngeal and nasal swab and wash	EUA 1/5/2020	[40]
TRUPCR SARS-CoV-2 Kit	India 3B Blackbio Biotech India Kilpest India subsidiary	RT-qPCR	10 copies/µL	Nasopharyngeal and oropharyngeal swabs, anterior nasal swab, and mid-turbinate nasal swabs, nasopharyngeal aspirates/washes or nasal aspirates, and bronchoalveolar lavage	EUA 18/6/2020	[41]
SARS-CoV-2 and Influenza A and B RT-qPCR Detection Kit	China 3D Medicines	RT-qPCR	5 copies/reaction	-	CE mark 3/2020	[42]
Abbott RealTime SARS-CoV-2 EUA test	USA Abbott	RT-qPCR	0.1 copies/µL	Nasopharyngeal swabs, Oropharyngeal swabs	EUA 18/3/2020	[43]
CareStart COVID-19 MDx RT-PCR	USA Access Bio	RT-qPCR	5.4 NDU/µL	Nasopharyngeal, oropharyngeal and nasal swabs, and nasopharyngeal wash/aspirate or nasal aspirate and bronchoalveolar lavage	EUA 7/7/2020	[44]
Acupath COVID-19 Real-Time (RT-PCR) Assay	USA Acupath Laboratories	RT-qPCR	25 copies/µL	Nasopharyngeal swab, nasopharyngeal aspirate, and bronchoalveolar lavage	EUA 29/6/2020	[45]
UltraGene Combo2Screen SARS-CoV-2 assay	Luxemburg Advanced Biological Laboratories	RT-qPCR	10E-6 TCID50/mL	Nasopharyngeal swab	EUA submission pending, CE mark 5/2020	[46]
RealStar SARS-CoV-2 RT-PCR Kits (1.0 and U.S versions)	Germany Altona Diagnostics	RT-qPCR	1.0E-01 PFU/mL	Human respiratory swabs	EUA 22/4/2020, CE mark 4/2020	[47]
Altru Dx SARS-CoV-2 RT-PCR assay	USA Altru Diagnostics	RT-qPCR	0.625 copies/µL	Nasal, midturbinate, nasopharyngeal, and oropharyngeal swab	EUA 30/4/2020	[48]
Bosphore Novel Coronavirus (2019-nCoV) Detection Kit	Turkey Anatolia Geneworks	RT-qPCR	-	Human respiratory sample	CE mark 2/2020	[49]
BioCode SARS-CoV-2 Assay	USA Applied BioCode	RT-qPCR	1.7E-2 TCID50/mL	Nasopharyngeal swabs (NPS), oropharyngeal swabs (OPS), nasal swabs or bronchoalveolar lavage	EUA 6/15/2020	[50]
Linea COVID-19 RT-PCR test	USA Applied DNA Sciences	RT-qPCR	1.25 copies/µL	Nasopharyngeal and oropharyngeal swabs, mid-turbinate nasal swabs, nasopharyngeal washes/aspirates or nasal aspirates, and bronchoalveolar lavage	EUA 5/13/2020	[51]
Aspirus SARS-CoV rRT-PCR Assay	USA Aspirus Reference Laboratory	RT-qPCR	0.5 copies/µL	Nasal, mid-turbinate, nasopharyngeal, oropharyngeal swab specimens and bronchoalveolar lavage specimens	EUA 6/1/2020	[52]
Assurance SARS-CoV-2 Panel	USA Assurance Scientific Laboratories	RT-qPCR	9 copies/µL	Nasal, nasopharyngeal, or oropharyngeal swabs	EUA 5/15/2020	[53]
A*STAR Fortitude 2.0	Singapore A*STAR, Tan Tock Seng Hospital of Singapore	RT-qPCR	25 copies/reaction	Nasal pharyngeal swab	Singapore Health Sciences Authority provisional authorization	[54]
Avera Institute for Human Genetics SARS-CoV-2 Assay	USA Avera Institute for Human Genetics	RT-qPCR	1.6 copies/µL	Nasopharyngeal, nasal, and oropharyngeal swab specimens	EUA 5/22/2020	[55]
Avellino SARS-CoV-2/COVID-19 (AvellinoCoV2)	USA Avellino Lab	RT-qPCR	18 NDU/µL	Nasopharyngeal swab and oropharyngeal swab	EUA 3/25/2020	[56]
Viro-Q SARS-CoV-2 kit	Germany BAG Diagnostics	RT-qPCR	-	-	CE mark 4/2020	[57]
ThermoFisher—Applied Biosystems TaqPath COVID-19 Combo Kit	USA Rutgers University Clinical Genomics Laboratory	RT-qPCR	0.250 GCE/µL	Nasopharyngeal swabs, nasopharyngeal aspirate (nasal aspirate), and nasopharyngeal aspirate (nasal aspirate), and bronchoalveolar lavage (BAL)	EUA 4/10/2020	[58]
Cobas SARS-CoV-2 Test	USA Roche	RT-qPCR	0.003 TCID50/mL	Nasopharyngeal and oropharyngeal swab samples	EUA 3/12/2020, CE mark 2020	[59]
GSD NovaPrime SARS-CoV-2 (COVID-19) Real-Time PCR test	Hungary Gold Standard Diagnostics/Eurofins Technologies	RT-qPCR	3.75 copies/reaction	Nasal wash/swab, nasopharyngeal wash/swab, oropharyngeal swab and bronchoalveolar lavage	CE mark 5/2020	[60]
QiaStat-Dx Respiratory SARS-CoV-2 Panel	USA Qiagen	RT-qPCR	180 NDU/µL	Nasopharyngeal swab	under CDC’s EUA	[61]
Rida Gene SARS-CoV-2	Germany R-Biopharma	RT-qPCR	50 copies/reaction	Human throat and nasopharyngeal swabs	CE mark 5/2020	[62]
Standard M nCoV Real-Time Detection Kit	Korea SD Biosensor	RT-qPCR	0.25 copies/μL	Nasoropharyngeal, nasal, and mid-turbinate nasal swab, and sputum	EUA 4/23/2020	[63]
ExProbe SARS-CoV-2 Testing Kit	Taiwan TBG Biotechnology	RT-qPCR	-	Nasopharyngeal and oropharyngeal swabs, anterior nasal and mid-turbinate nasal swabs	EUA 6/10/2020	[64]
Quick SARS-CoV-2rRT-PCR Kit	USA Zymo Research	RT-qPCR	2.5 E3 GEC/μL	Nasal, nasopharyngeal, mid-turbinate or oropharyngeal swabs), and lower respiratory specimens (such as sputum, tracheal aspirates, and bronchoalveolar lavage)	EUA 5/7/2020	[65]
Fulgent COVID-19 by RT-PCR Test	USA Fulgent Genetics/Fulgent Therapeutics	RT-qPCR	20 copies/µL	Nasal, nasopharyngeal, and oropharyngeal swabs	EUA 5/15/2020	[66]
SARS-CoV-2 RT-PCR test	Germany Centogene	RT-qPCR	5 copies/µl	Dry oropharyngeal swabs	EUA 7/1/2020	[67]
Xpert Xpress SARS-CoV-2 test	USA Cepheid	RT-qPCR	0.0001 PFU/µL	Nasopharyngeal, oropharyngeal, nasal, or mid-turbinate swab and/or nasal wash/aspirate	EUA 3/20/2020	[68]
HDPCR SARS-CoV-2 real-time PCR assay	USA ChromaCode	RT-qPCR	0.250 copies/µL	Nasopharyngeal swabs oropharyngeal swabs, anterior nasal swabs, midturbinate nasal swabs, nasal aspirate, nasal wash, and bronchoalveolar lavage (BAL) specimens	EUA 6/9/2020	[69]
Hymon SARS-CoV-2 Test Kit	China Dba SpectronRx	RT-qPCR	1.2 copies/µl	Nasal, mid-turbinate, nasopharyngeal, and oropharyngeal swab specimens)	EUA 5/22/2020	[70]
DSL COVID-19 Assay	USA Diagnostic Solutions Laboratory	RT-qPCR	18 NDU/µL	Nasopharyngeal swab	EUA 6/25/2020	[71]
Simplexa COVID-19 Direct	USA DiaSorin Molecular	RT-qPCR	6 NDU/µL	Nasal swab, nasopharyngeal swab, nasal wash/aspirate, and BAL	EUA 3/19/2020, CE mark 4/2020	[72]
Lilly SARS-CoV-2 Assay	USA Eli Lilly	RT-qPCR	1 copy/µL	Nasopharyngeal swabs, oropharyngeal (throat) swabs, anterior nasal swabs, mid-turbinate nasal swabs, nasal aspirates, nasal washes and bronchoalveolar lavage	EUA 7/27/2020	[73]
Ampiprobe SARS-CoV-2 Test System	USA Enzo Biochem/Enzo Life Sciences	RT-qPCR	0.280 copies/µL	Nasopharyngeal swabs	EUA 7/7/2020	[74]
Euroimmun/PerkinElmer	USA EuroRealTime SARS-CoV-2	RT-qPCR	0.150 copies/µL	Nasal, mid-turbinate, nasopharyngeal, oropharyngeal swabs and bronchioalveolar lavage	EUA 6/8/2020, CE mark 3/2020	[75]
Fosun COVID-19 RT-PCR Detection Kit	China Fosun Pharma USA	RT-qPCR	0.3 copies/µL	Anterior nasal swabs, mid-turbinate nasal swabs, nasopharyngeal swabs, oropharyngeal swabs, sputum, lower respiratory tract aspirates, bronchoalveolar lavage, and nasopharyngeal wash/aspirate or nasal aspirate	EUA 4/17/2020	[76]
NeoPlex COVID-19 Detection Kit	Korea GeneMatrix	RT-qPCR	5.4 NDU/µL	Nasopharyngeal swabs	EUA 5/14/2020	[77]
GB SARS-CoV-2 Real-Time RT-PCR	Taiwan General Biologicals	RT-qPCR	0.1 copies/µL	Nasopharyngeal swabs	CE mark 4/2020	[78]
Genetron SARS-CoV-2 RNA Test	China Genetron	RT-qPCR	10 copies/µL	Oropharyngeal, nasopharyngeal, anterior nasal and mid-turbinate nasal swab	EUA 6/5/2020	[79]
Helix COVID-19 test	USA Helix	RT-qPCR	1 GCE/µL	Nasopharyngeal and oropharyngeal swabs	EUA 7/23/2020	[80]
Panther Fusion SARS-CoV-2 assay	USA Hologic	RT-qPCR	1 × 10^−5^ TCID50/µL	Nasopharyngeal (NP), nasal, oropharyngeal (OP) swab specimens and lower respiratory tract (LRT) specimens	EUA 3/16/2020	[81]
Smart Detect SARS-CoV-2 rRT-PCR Kit	USA InBios International	RT-qPCR	12 GCE/reaction	Nasopharyngeal swab, anterior nasal swab and mid-turbinate nasal swab	EUA 4/7/2020	[82]
IDT 2019-novel coronavirus kit	USA Integrated DNA Technologies/Danaher	RT-qPCR	-	Oropharyngeal, nasopharyngeal, anterior nasal and mid-turbinate nasal swab	under CDC’s EUA	[83]
SARS-CoV-2 Assay	USA Integrity Laboratories	RT-qPCR	2.5 copies/µL	Nasal, nasopharyngeal and oropharyngeal swab	EUA 4/13/2020	[84]
ProTect Covid-19 kit	Singapour JN Medsys	RT-qPCR	<2%	Upper respiratory nasopharyngeal swabs	Under CDC’s EUA, CE mark 4/2020, Singapore HAS provisional authorization; Philippines FDA	[85]
COVID-19 Coronavirus Real Time PCR Kit	China Jiangsu Bioperfectus Technologies	RT-qPCR	0.350 copies/µL	Nasopharyngeal swabs, oropharyngeal (throat) swabs, anterior nasal swabs, mid-turbinate nasal swabs, nasal aspirates, nasal washes, bronchoalveolar lavage (BAL) fluid and sputum	EUA 18/6/2020	[86]
Curative-Korva SARS-Cov-2 Assay	USA KorvaLabs	RT-qPCR	0.200 copies/µL	Oropharyngeal (throat) swab, nasopharyngeal swab, nasal swab, and oral fluid specimens	EUA 16/4/2020	[87]
Idylla SARS-CoV-2 Test	Belgium Biocartis	RT-qPCR	0.5 copies/µL	Nasopharyngeal swab	CE mark 11/2020	[88]
BioFire Respiratory Panel 2.1-EZ (RP2.1-EZ)	USA BioMérieux/BioFire Diagnostics	RT-qPCR	6 NDU/µL	Nasopharyngeal swab	EUA 2/10/2020	[89]
VitaPCR Influenza A and B/SARS-CoV-2 assay	Singapore Credo Diagnostics Biomedical	RT-qPCR	2.73 copies/μl	Nasopharyngeal swab	CE mark 10/2020	[90]
Genetworx Covid-19 Nasal Swab Test	USA Genetworx	RT-qPCR	0.274 copies/µL	Nasal swab	EUA 15/12/2020	[91]
EuroRealTime SARS-CoV-2/Influenza A/B	Germany Euroimmun/PerkinElmer	RT-qPCR	1.8 NDU/µL	Throat swab	CE mark 12/2020	[92]
MassArray SARS-CoV-2 Panel	USA Agena Bioscience	RT-PCR/MALDI-TOF	0.3 copies/µL	Nasopharyngeal swab, oropharyngeal swab, and BAL	EUA 10/26/2020 CE mark 9/2020	[93]
Bio-Rad SARS-CoV-2 ddPCR Test	USA Bio-Rad Laboratories	ddPCR	0.625 copies/µL	Nasopharyngeal, anterior nasal and mid-turbinate swab specimens as well as nasopharyngeal wash/aspirate and nasal aspirate specimens	EUA 1/5/2020	[94]
iAMP COVID-19 Detection Kit	USA Atila BioSystems	Isothermal amplification (OMEGA amplification)	10 copies/µL	Nasal, nasopharyngeal (NP), and oropharyngeal (OP) swabs	EUA 10/4/2020	[95]
ID Now COVID-19	USA Abbott	Isothermal amplification (proprietary enzymes)	0.125 GCE/µL	Direct nasal, nasopharyngeal or throat swabs	EUA 27/3/2020	[96]
Color SARS-CoV-2 LAMP Diagnostic Assay	USA Color	RT-LAMP	0.75 copies/µL	Nasopharyngeal (NP) swabs, oropharyngeal (OP) swabs, anterior nares (AN) swabs, mid-turbinate nasal (MTN) swabs, NP wash/aspirate or nasal aspirates, and bronchoalveolar lavage specimens	EUA 5/18/2020, amended 24/7/2020	[97]
2019-nCoV detection kit	China Rendu Biotechnology	RT-LAMP	-	Nasal, nasopharyngeal, and oropharyngeal swab	China NMPA 3/2020	[98]
AQ-TOP COVID-19 Rapid Detection Kit	Korea Seasun Biomaterials	RT-LAMP	7 copies/µL	Oropharyngeal and nasopharyngeal swab specimens, anterior nasal and mid-turbinate nasal swabs, nasopharyngeal wash/aspirate or nasal aspirate specimens, bronchoalveolar lavage (BAL) and sputum from individuals	EUA 21/5/2020	[99]
Lucira Health	USA Lucira COVID-19 All-In-One Test Kit	RT-LAMP	0.9 copies/µL	Nasal swab	EUA 17/11/2020	[100]
Poplar SARS-CoV-2 TMA Pooling assay	USA Poplar Healthcare	TMA	-	Nasal, nasopharyngeal, and oropharyngeal swab	EUA 3/8/2020	[101]
COVIDSeq Test	USA Illumina	Next generation gene sequencing	5.4 NDU/µL	Nasopharyngeal (NP), oropharyngeal (OP), and mid-turbinate (MT) nasal swabs	EUA 9/6/2020	[102]
Fulgent COVID-19 by NGS	USA Fulgent Genetics/MedScan Laboratory	Next generation gene sequencing	3.6 NDU/µL	Nasal, nasopharyngeal, and oropharyngeal swabs	EUA submission pending	[103]
Sherlock CRISPR SARS-CoV-2 kit	USA Sherlock Biosciences	RT-LAMP and CRISPR-Cas 13	6.75 copies/µL	Nasopharyngeal and oropharyngeal swab samples	EUA 6/5/2020	[104]
SARS-CoV-2 RNA DETECTR Assay	USA UCSF Clinical Labs at China Basin	CRISPR-Cas12	20 copies/µL	Nasopharyngeal swabs, oropharyngeal (throat) swabs, mid-turbinate nasal swabs, anterior nasal swabs, nasopharyngeal wash/aspirate or nasal aspirate	EUA 9/7/2020	[105]

LoD: Limit of detection. TCID50: Median Tissue Culture Infectious Dose. GCE: Genome copy equivalents. NDU: RNA NAAT detectable units. PFU: plaque-forming unit.

**Table 2:** Serological tests that have some certification to date, FDA. Include: % sensitivity, %, specificity, cross reactivity, interferences, mark, country.

Diagnostic	Country Manufacturer	Tech	Specificity	Sensitivity	Cross Reactivity	Interferences	Date EUA Issued	Ref
Babson Diagnostics aC19G1	USA Babson Diagnostics, Inc.	IgG CLIA	100%	100%	Anti-HIV 1 + 2, Anti-HCV, CMV IgG, Anti-HBs, Anti-HAV EIA	N/A	23/6/2020	[196]
Access SARS-CoV-2 IgG	USA Beckman Coulter, Inc.	IgG CLIA	99.6%	96.8%	Human chorionic gonadotropin (hCG), HIV antibody, Influenza antibody, Influenza A antibody, Influenza B antibody, Measles antibody, Mycoplasma pneumoniae IgG, Parvovirus B19 antibody, Respiratory pathogen antibodies, Respiratory syncytial virus (RSV) antibody	Hemoglobin, Bilirubin (conjugated), Bilirubin (unconjugated), Triglycerides (Intralipid)	26/6/2020	[197]
Diazyme DZ-Lite SARS-CoV-2 IgG CLIA Kit	USA, Diazyme Laboratories, Inc.	IgG CLIA	97.4%	100%	Influenza A H1N1 IgM/IgG, Influenza A H7N9 IgM/IgG, Rhinovirus Type A IgM/IgG, Rotavirus IgM/IgG, Human coronavirus HKU1 IgM/IgG, Human coronavirus NL63 IgM/IgG, ANA	Triglycerides, Hemoglobin, Rheumatoid Factor, Anti-Mitochondrial, HAMA, Total IgG, Total IgM, Interferon α, Ribavirin	8/7/2020	[198]
SCoV-2 Detect IgG ELISA	USA, InBios International, Inc.	IgG ELISA	98.9%	97.8%	VIH 1 y 2, hepatitis C and B	Hemoglobin Bilirubin Triglycerides Cholesterol	10/6/2020	[199]
SARS-CoV-2 RBD IgG test	USA, Emory Medical Laboratories	IgG ELISA	96.4%	100%	Anti-Influenza B, Anti-HCV, Anti-HBV, Anti-Haemophilus, Anti-Rhinovirus influenzae, ANA, Anti-HIV	N/A	15/6/2020	[200]
xMAP SARS-CoV-2 Multi-Antigen IgG Assay	USA, Luminex Corporation	IgG FMIA	99.3%	96.3%	N/A	(dipotassium EDTA)	16/7/2020	[201]
ADVIA Centaur SARS-CoV-2 IgG (COV2G)	Germany, Siemens Healthcare Diagnostics Inc.	IgG Semi-quantitative	99.9%	100%	Chlamydia trachomatis IgM, Cytomegalovirus (CMV) IgM, Epstein Barr virus (EBV) IgG, Epstein Barr virus (EBV) IgM, Hepatitis A virus (HAV) IgM, Hepatitis B core (Anti-HBc) IgM, Hepatitis B core (Anti-HBc) total antibody, Hepatitis C virus (HCV) antibody, Herpes simplex virus (HSV) IgM	Hemoglobin, Bilirubin (conjugated), Bilirubin (unconjugated), Triglycerides (Intralipid), Biotin	31/7/2020	[202]
Atellica IM SARS-CoV-2 IgG (COV2G)	Germany, Siemens Healthcare Diagnostics Inc.	IgG Semi-quantitative	99.9%	100%	Human chorionic gonadotropin (hCG), Human immunodeficiency virus (HIV) antibody, Influenza antibody, Influenza A antibody, Influenza B antibody, Measles antibody	Hemoglobin, Bilirubin (conjugated), Bilirubin (unconjugated), Triglycerides (Intralipid), Biotin, Cholesterol, Protein, total	7/31/2020	[203]
Anti-SARS-CoV-2 ELISA (IgG)	Germany, EUROIMMUN US Inc.	Serology IgG	99.6%	86.7%	Influenza, Acute EBV infection, Rheumatoid factor, other Human coronavirus	Hemoglobin, triglycerides, and bilirubin	4/5/2020	[204]
SARS-CoV-2 IgG assay	USA, Abbott Laboratories Inc.	Serology IgG	99%	100%	CMV, Hepaitis A,B, RSV, Rubella, Herpes virus	Adenovirus, pregnant female, lupus	26/4/2020	[205]
LIAISON SARS-CoV-2 S1/S2 IgG	Italy, DiaSorin Inc.	Serology IgG	99.3%	97.6%	Anti-Human CoV OC43; Anti-Human CoV HKU1, 4 Anti-Human CoV unknown strain.	Triglycerides, Hemoglobin, Unconjugated bilirubin	24/4/2020	[206]
VITROS Immunodiagnostic Products Anti-SARS-CoV-2 IgG Reagent Pack	USA, Ortho-Clinical Diagnostics, Inc.	Serology IgG	100%	83.3%	Adenovirus Antibody, Influenza A IgG, Influenza A IgM, Influenza B IgG, Influenza B IgM, Coxsackie Virus Antibody, Echovirus Antibody, Polio Virus, Anti-respiratory syncytial virus (RSV), HCV Antibody, Anti Nuclear Antibody	Bilirubin (conjugated), Bilirubin (unconjugated), Biotin, Hemoglobin, Intralipid	24/4/2020	[207]
COVID-19 ELISA IgG Antibody Test	USA, Mount Sinai Laboratory	Serology IgG	100%	92%	Varicella, Infleunza, Hepatitis, HIV, CMV	Ascorbic Acid, Hemoglobin, Bilirubin, Albumin, Triglyceride	15/4/2020	[208]
SCoV-2 Detect IgM ELISA	USA, InBios International, Inc.	IgM ELISA	100%	100%	Anti-Influenza A/B, Anti-Hepatitis B, Anti-Hepatitis C, Anti-Nuclear Antibody, Rheumatoid Factor, Human Anti-Mouse Antibody, Anti-HIV, Anti-Respiratory Syncytial Virus, Normal Human Sera	Hemoglobin, Bilirubin (conjugated and unconjugated), Triglycerides, Buffer (SDB), Cholesterol	30/6/2020	[199]
VIDAS SARS-CoV-2 IgM	France, bioMérieux SA	IgM ELFA	99.4%	100%	SARS-CoV(-1) Infection (2005), SARS-CoV(-1) Infection (2020), HCoV-NL63 Infection, HCoV-229E Infection, HCoV-OC43 Infection HCoV-HKU1 Infection MERS-CoV Infection, Acute EBV infections with heterophile antibodies, ANA	Hemoglobin, Lipids, Albumin, Bilirubin (conjugated), Bilirubin (unconjugated)	6/8/2020	[209]
RightSign COVID-19 IgG/IgM Rapid Test Cassette	China, Hangzhou Biotest Biotech Co., Ltd.	IgM and IgG Lateral Flow	100%	93.3%	Anti-FLU A, Anti-FLU B, Anti-Respiratory Syncytial, Virus Anti-Adenovirus, Anti-HBsAg, Anti-Syphilis, Anti-H. Pylori, Anti-HIV, Anti-HCV, Anti-SARS-COV	Acetaminophen, Caffeine, Albumin, Acetylsalicylic Acid, Gentisic Acid, Ethanol, Ascorbic Acid	4/6/2020	[210]
LYHER Novel Coronavirus (2019-nCoV) IgM/IgG Antibody Combo Test Kit (Colloidal Gold)	China, Hangzhou Laihe Biotech Co., Ltd.	IgM and IgG Lateral Flow	98.8%	96.7%	H1N1-1~H1N1-3, H7N9-1~H7N9-2, ANA-1, Staphyl. -1~Staphyl.-2, EBV-1~EBV-5, RSV-1~RSV-2, Chlamydia-1~Chlamydia-3	N/A	19/6/2020	[211]
Assure COVID-19 IgG/IgM Rapid Test Device	China, Assure Tech. (Hangzhou Co., Ltd.)	IgM and IgG Lateral Flow	100%	98.8%	Human coronavirus HKU1 IgM/IgG, Human coronavirus NL63 IgM/IgG	Icteric (Bilirubin), Lipemicicines Acetylsalicylic acid Ascorbic acid (Vitamin C), Amoxicillin	6/7/2020	[212]
Sienna-Clarity COVIBLOCK COVID-19 IgG/IgM Rapid Test Cassette	Finland, Salofa Oy	IgM and IgG Lateral Flow	99.2%	94.9%	HCV, HBV, ANA Metapneumovirus	Ascorbic Acid, Hemoglobin, Bilirubin, Albumin, Triglyceride	13/7/2020	[213]
Rapid COVID-19 IgM/IgG Combo Test Kit	USA, Megna Health, Inc.	IgM and IgG Lateral Flow	97.5%	100%	Influenza A Virus, Influenza B Virus, Adenovirus, Rotavirus and Mycoplasma Pneumoniae.	Rheumatoid Factor, Bilirubin, Triglyceride, Hemoglobin	17/7/2020	[211]
CareStart COVID-19 IgM/IgG	USA, Access Bio, Inc.	IgM and IgG Lateral Flow	100%	98.8%	Anti-Influenza A, Anti-HCV, Anti-229E (alpha coronavirus), Anti-OC43 (beta coronavirus), Anti-HKU1 (beta coronavirus), Antinuclear antibodies (ANA), Anti-respiratory syncytial virus, Anti-Rhinovirus	Acetaminophen, HAMA, Acetylsalicylic acid, Hemoglobin, Albendazole, Ibuprofen, Chloroquine diphosphate, Rifampicin	24/7/2020	[214]
BIOTIME SARS-CoV-2 IgG/IgM Rapid Qualitative Test	China, Xiamen Biotime Biotechnology Co., Ltd.	IgM and IgG Lateral Flow	98.8%	100%	Anti-OC43 (beta coronavirus), Anti-HKU1 (beta coronavirus), Antinuclear antibodies (ANA)	N/A	24/7/2020	[215]
Vibrant COVID-19 Ab Assay	USA, Vibrant America Clinical Labs	IgM and IgG CLIA	98.3%	97.1%	Anti-Influenza A, Anti- Influenza B, Anti-HCV, Anti-HBV, ANA, Anti-respiratory syncytial virus, Anti-Haemophilus influenzae	Bilirubin, Triglycerides Hemoglobin Rheumatoid Factor (RF) Cholesterol, HAMA, Ribavirin, Levofloxacin, Azithromycin, Ceftriaxone sodium	4/6/2020	[216]
Anti-SARS-CoV-2 Rapid Test	China, Autobio Diagnostics Co. Ltd.	Serology IgM and IgG	97%	93%	Other human coronavirus, Influenza	HAMA positive sample, Rheumatoid factor, Antinuclear antibody (ANA), Anti-mitochondrial antibody (AMA), Bilirubin	24/4/2020	[217]
DPP COVID-19 IgM/IgG System	Usa, Chembio Diagnostic System, Inc.	Serology IgM and IgG	100%	95%	Human coronavirus, Zika, Influenza A, B, Mononucleosis, Chikungunya, Yellow fever virus, Dengue virus	Biotin Hemoglobin	14/4/2020	[218]
qSARS-CoV-2 IgG/IgM Rapid Test	USA, Cellex Inc.	Serology IgM and IgG	96.0%	93.8%	Human coronavirus, Adenovirus, Influenza A, B, Rhinovirus, Chlamydia pneumoniae, Streptococcus pneumoniae, Mycobacterium tuberculosis, Mycoplasma pneumoniae	Hemoglobin, Bilirubin (conjugated), Triglycerides, Cholesterol	1/4/2020	[219]
COVID-19 IgG/IgM Rapid Test Cassette (Whole Blood/Serum/Plasma)	USA, Healgen Scientific LLC	Serology IgM and IgG	96.7%	97%	Influenza A virus IgG, Influenza B virus IgG, Respiratory syncytial virus IgG, Adenovirus IgG, Rhinovirus IgG, Human metapneumovirus IgG, Mycoplasma pneumoniae IgG, Chlamydia pneumoniae IgG, HCV IgG, Haemophilus influenza IgG, HBV core antibody IgG	Ascorbic Acid, Hemoglobin, Bilirubin, Albumin, Triglyceride	29/5/2020	[210]
Dimension EXL SARS-CoV-2 Total antibody assay (CV2T)	USA, Siemens Healthcare Diagnostics Inc.	Total Antibody CLIA	99.9%	100%	Anti-influenza A, Anti-influenza B, AntiHBV Antinuclear, antibody (ANA), Hepatitis B core antigen (anti-HBc) IgM, Hepatitis B surface antigen (HBs Ag), Hepatitis C virus (HCV) antibody, HIV antibody, influenza antibody	Hemoglobin, Bilirubin, conjugated, Bilirubin, Lipemia (Intralipid)	8/6/2020	[220]
WANTAI SARS-CoV-2 Ab ELISA	China, Beijing Wantai Biological Pharmacy Enterprise Co., Ltd.	Total Antibody ELISA	100%	94.5%	HBsAg and antibodies to HIV 1/2, HCV, TP	N/A	5/8/2020	[221]
WANTAI SARS-CoV-2 Ab Rapid Test	China, Beijing Wantai Biological Pharmacy Enterprise Co., Ltd.	Total Antibody Lateral Flow	98.8%	100%	alpha COV 229E, alpha COV NL63, beta COV OC43, beta COV HKU1 Flu A, Flu B, HCV, HBV, ANA	N/A	10/7/2020	[222]
New York SARS-CoV Microsphere Immunoassay for Antibody Detection	USA, Wadsworth Center, New York State Department of Health	Serology Total Antibody	99.1%	99%	HCV, HIV, Measles, Mumps, Rubella, Varicella Zoster virus, WNV, Herpes Simplex virus, ZIKV, Enterovirus, Rheumatoid factor	Biotin Hemoglobin	30/4/2020	[223]
Platelia SARS-CoV-2 Total Ab assay	USA, Bio-Rad Laboratories, Inc.	Serology Total Antibody	98%	99%	Influenza, Mycoplasma pneumoniae, Rheumatoid factor, Other human coronavirus		29/4/2020	[224]
Elecsys Anti-SARS-CoV-2	Germany, Roche Diagnostics	Serology Antibody	99.8%	100%	Other human coronavirus, Influenza	Biotin Hemoglobin	2/5/2020	[225]

**Table 3:** FDA-approved antigen-based diagnostic tests.

Diagnostic	Country, Manufacturer	Technology	Specificity	Sensitivity	Ref
Lateral Flow, Visual Read	USA, Ortho clinical Diagnostic, Inc.	Chemiluminescence immunoassay	98.50%	97.10%	[232]
LumiraDx SARS·CoV-2 Ag Test	Spain, LumiraDx UK Ltd.	Chromatographic Digital immunoassay	96.50%	98%	[233]
BD Veritor System for Rapid Detection	England, Becton Dickinson	Chromatographic immunoassay	100%	84%	[234]
Clip COVID Rapid Antigen Test	USA, Luminostics, Inc.	Lateral Flow, Immunoluminescent Antigen	100%	100%	[235]
CareStart COVID-19 Antigen test	USA, Access Bio, Inc.	Lateral Flow, Antigen	98%	99%	[44]
Ellume COVID-19 Home Test	Australia, Ellume Limited Test	Lateral Flow, Fluorescence	100%	100%	[236]
Sofia 2 SARS Antigen FIA	Germany, Quidel Corporation	Lateral Flow, Fluorescence Antigen	100%	80%	[237]
QuickVue SARS Antlgen Test	USA, Quidel Corporatlon	Lateral Flow, Visual Read	98%	98%	[238]
BinaxNOW COVID-19 Ag Card Home	USA, Abbott Diagnostics	Lateral Flow, Visual Read	98%	98%	[239]
BinaxNOW COVID-19 Ag card	USA, Abbott Diagnostics Scarborough, Inc.	Lateral Flow, Visual Read	98.50%	97.10%	[240]
Sofía 2 Flu+ SARS Antlgen FIA	Germany, Quidel Corporatlon	Lateral Flow, Fluorescence, Instrument Read, Multl-Analyte	100%	80%	[241]
Sampinute COVID-19 Antlgen MIA	USA, Celltrion, Inc.	Magnetic force assisted electrochemical	100%	100%	[242]
Simoa SARS-CoV-2 N Protein Antigen test	USA, Quanterix	Paramagnetic Microbead-based	98.50%	97.10%	[243]

**Table 4:** Nanoparticle-based tests to detect several coronaviruses.

Virus	NPs	Readout/Output	LoD	T (min)	Ref
SARS-CoV	AuNPs	Immunosensing	10 fmol	30	[253]
PEDV	AU NPs	Nano nest PCR	2.21 × 10^−7^ ngμl^−1^	45	[254]
Mers CoV	AgNPs	Colorimetric	1.53 nM	20	[255]
Mers CoV	AgNPs	Electrochemiluminescence	1.0 pg/mL^−1^	20	[256]
Mers CoV	S.aureus nanobioparticles	Agglutination test	0.93055556	20	[257]
IBV	MoS2 nanosheets	Immunosensing	4.6 × 10^2^ EID50 per mL	20	[258]
IBV	Qd-MP NPs and Zr NPs	Photoluminesce	79.15 EID/50 mL	20	[259]
IBV	CAu NPs	Chiroimmunosensing	47.91 EID/50 mL	20	[260]
IBV	Colloidal Au NPs	ICS	10 4.4 EID/50 mL	45	[257]
HCoV	AgNPs	Electrochemiluminescence	0.4 pgml^−1^	20	[257,261]
